# MCT-1 expression and PTEN deficiency synergistically promote neoplastic multinucleation through the Src/p190B signaling activation

**DOI:** 10.1038/onc.2014.125

**Published:** 2014-05-26

**Authors:** M-H Wu, Y-A Chen, H-H Chen, K-W Chang, I-S Chang, L-H Wang, H-L Hsu

**Affiliations:** 1 Institute of Molecular and Genomic Medicine, National Health Research Institutes, Taiwan, ROC; 2 Institute of Population Health Science, National Health Research Institutes, Taiwan, ROC; 3 National Institute of Cancer Research and Division of Biostatistics and Bioinformatics, National Health Research Institutes, Taiwan, ROC

## Abstract

Multinucleation is associated with malignant neoplasms; however, the molecular mechanism underlying the nuclear abnormality remains unclear. Loss or mutation of PTEN promotes the development of malignant tumors. We now demonstrate that increased expression of the oncogene MCT-1 (*m*ultiple *c*opies in *T*-cell malignancy 1) antagonizes *PTEN* gene presentation, PTEN protein stability and PTEN functional activity, thereby further promoting phosphoinositide 3 kinase/AKT signaling, survival rate and malignancies of the PTEN-deficient cells. In the PTEN-null cancer cells, MCT-1 interacts with p190B and Src *in vivo*, supporting that they are in proximity of the signaling complexes. MCT-1 overexpression and PTEN loss synergistically augments the Src/p190B signaling function that leads to inhibition of RhoA activity. Under such a condition, the incidence of mitotic catastrophes including spindle multipolarity and cytokinesis failure is enhanced, driving an Src/p190B/RhoA-dependent neoplastic multinucleation. Targeting MCT-1 by the short hairpin RNA markedly represses the Src/p190B function, improves nuclear structures and suppresses xenograft tumorigenicity of the PTEN-null breast cancer cells. Consistent with the oncogenic effects *in vitro*, clinical evidence has confirmed that *MCT-1* gene stimulation is correlated with *p190B* gene promotion and *PTEN* gene suppression in human breast cancer. Accordingly, *MCT-1* gene induction is recognized as a potential biomarker of breast tumor development. Abrogating MCT-1 function may be a promising stratagem for management of breast cancer involving Src hyperactivation and/or PTEN dysfunction.

## Introduction

Loss-of-function mutations in the catalytic domain of PTEN or the reduced PTEN expression through loss of heterozygosity has been identified in human cancers and inherited cancer-predisposition syndromes.^1, 2, 3, 4, 5^ PTEN inhibits phosphoinositide 3 kinase (PI3K)/AKT signaling pathway.^6^ A subtle decrease in PTEN amount (80% of normal levels) induces tumorigenicity, particularly in breast cancer.^7^ *PTEN* gene is methylated in ductal carcinoma *in situ* and in early invasive breast cancer, indicating the epigenetic inactivation of PTEN during cancer progression.^8^ NEDD4-1 catalyzes PTEN polyubiquitination and degradation decreasing the cytoplasmic PTEN in carcinogenesis.^9^ However, PTEN monoubiquitination enhances its nuclear import and antitumor effect perhaps by preventing nuclear AKT activity and genomic instability.^10,11^

Temporal and spatial distribution of the PI3K regulates cytokinesis.^12^ PI3K and PTEN function at spindle poles and cleavage furrow in mitosis, respectively. Loss of PTEN deregulates the PI(3,4,5)P3 production increasing the frequency of cytokinesis failure and multinucleation. The nuclear–cytoplasmic shuttling of PTEN also modulates cell cycle and apoptosis.^13^ Cytoplasmic PTEN dephosphorylates AKT, upregulates p27(kip1) and induces apoptosis. Nuclear PTEN reduces cyclin D1 expression and mitogen-activated protein kinase activity, thus interfering with cell cycle progression. Nuclear PTEN also maintains chromosomal stability via induced Rad51 and DNA damage repair.^14,15^ Under oxidative stress, PTEN accumulated in the nucleus increases p53 function that prevents genotoxicity and tumor growth.^16^

The p190A has been reported to  accumulate temporally at the contracting cleavage furrow and reduce in late mitosis by ubiquitin–proteasome degradation.^17, 18, 19^ Overexpressing p190A decreases the active RhoA-GTP levels at cleavage furrow, leading to cytokinesis failure and multinucleation. The phosphorylated p190B at tyr1109 residue, which corresponds to an Src consensus target site on p190A, is potentially required for mitotic progression.^20^ Therefore, deregulated p190B expression increases the events of aneuploidy, chromosome miss-segregation and apoptosis. The PI3K catalytic subunit (p110delta) stimulates p190A that inactivates RhoA and PTEN function,^21^ whereas the inhibition of p110delta suppresses p190A, resulting in the activation of RhoA and PTEN. The stability and activity of PTEN are regulated by phosphorylation at the C-terminal tail (ser380, thr382 and thr383) such as CK2-induced phosphorylation at the C-terminal position induces PTEN degradation.^22,23^ Src-phosphorylated PTEN also causes PTEN degradation and PI3K/AKT signaling amplification.^24^ In an inhibitory loop, PTEN dephosphorylates Src at tyr416 residue to inactivate Src.^25^ Thus, Src is highly activated in PTEN-deficient cells.

MCT-1 (*m*ultiple *c*opies in *T*-cell malignancy 1) oncogene stimulates Ras and AKT signaling function.^26, 27, 28^ Similar to PTEN,^14^ MCT-1 relocates from the cytoplasm to the nucleus upon genotoxicity.^29^ In support of MCT-1 oncogenic role in genomic instability, MCT-1 suppresses p53 activity and increases the frequency of massive chromosomal aberrations upon DNA damage.^27,29^ Depletion of p53 enhances the MCT-1 oncogenic effect on chromosomal destabilization, mitotic abnormality and tumor growth,^27,28,30^ implying an antagonism between p53 and MCT-1 in the neoplastic progression.

In this study, we identified a novel inhibitor of PTEN and the interacting protein of Src/p190B, MCT-1, and demonstrated that PTEN loss and MCT-1 induction synergistically promoted the acytokinetic division and neoplastic multinucleation via the Src/p190B signaling activation. Targeting MCT-1 in the PTEN-null cancer cells improved the mitotic checkpoint and nuclear integrity, but suppressed tumor growth. The clinical studies confirm that MCT-1 is frequently overexpressed together with p190B upregulation and PTEN downregulation in human breast cancers.

## Results

### Overexpression of MCT-1 destabilizes PTEN

MCT-1 oncogene induces the AKT phosphorylation (ser473).^26^ To investigate if MCT-1 inhibits PTEN to activate AKT, the MCF-10A cells were starved for 24 h followed by the serum activation for 30 min (Supplementary Figure S1a). We observed that the phosphorylated PTEN (ser380) (2.3-fold) and AKT (ser473) (95.5-fold) were much enhanced in the ectopic MCT-1-expressing condition (lane 5) than in the control cells. PTEN is inactivated upon ser380 phosphorylation,^23^ suggesting that MCT-1 inhibits PTEN via the posttranslational modification. Consistent with wortmannin and LY294002 (LY) suppress PI3K/AKT signaling,^31^ LY inhibited the MCT-1-induced AKT activation (Supplementary Figure S1a, lane 6) and wortmannin suppressed the MCT-1-stimulated AKT in the MCF-7 cells (Supplementary Figure S1b, lane 6). The epidermal growth factor (EGF)-induced AKT phosphorylation was also markedly suppressed by LY and attenuated by an Src inhibitor (PP2) in the MCT-1-expressing MCF-10A cells (Supplementary Figure S1c, lanes 9 and 10), showing the involvement of Src and PI3K in the MCT-1 pathway.

**Figure 1:**
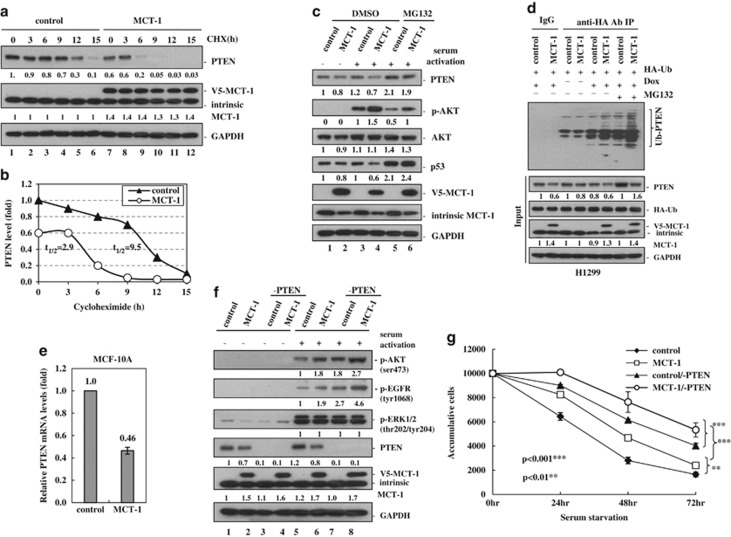
MCT-1 decreases PTEN expression and increases cell viability. MCF-10A cells without (control) or with (MCT-1) MCT-1 overexpression were examined. (**a**) The cells were treated with 200 μM cyclohexamide (CHX) for the indicated time points. PTEN degradation was analyzed. (**b**) PTEN half-life in the control and ectopic MCT-1 cells were indicated. (**c**) The levels of PTEN, p53 and active-phospho-AKT (ser473) were examined without (dimethylsulfoxide (DMSO)) or with MG132 treatment. (**d**) An *in vivo* ubiquitination assay was conducted. The H1299/TR cells were induced by doxycycline and transfected with the vector encoding HA-ubiquitin. The ubiquitinated PTEN (Ub-PTEN) was IP with anti-HA Ab and detected by PTEN Ab. (**e**) *PTEN* mRNA levels were analyzed by quantitative real-time polymerase chain reaction. (**f**) The active phosphorylated AKT and EGFR were analyzed in the MCF-10A cells with MCT-1 expression and PTEN knockdown (MCT-1/−PTEN) and compared with the control cells depleting PTEN (control/−PTEN) and the PTEN-proficient cells (control, MCT-1) upon serum activation for 30 min after 24 h starvation. (**g**) The cells were starved for various time and surviving cells were determined by MTT (3-(4, 5-dimethylthiazol-2-yl)-2, 5-diphenyltetrazolium bromide) assay. **p<0.01 and ***p<0.001.

The steady state of PTEN determines inhibitory effect on PI3K. PTEN stability was examined by blocking protein biosynthesis with cyclohexamide in the MCF-10A cells (Figure 1a). At different intervals, the remaining PTEN amounts were quantified by densitometry, normalized to glyceraldehyde 3-phosphate dehydrogenaseand compared with the initial PTEN level (at time 0). Results showed that PTEN had a longer half-life (9.5 h) in the control cells than the MCT-1-increasing cells (2.9 h) (Figure 1b). Similar results were observed in the MDA-MB-231 cells treated with cyclohexamide for different periods that MCT-1 expression promoted PTEN degradation and, therefore, shortening PTEN half-life (6.2 h) relative to the control cells (7.6 h) (Supplementary Figures S1d and e). To examine if MCT-1 mediated PTEN reducing by proteasome, the MCF-10A cells were starved, treated with or without MG132 and reactivated by the serum (Figure 1c). Taken together with increased p53 stability, MG132 elevated PTEN level and stability in the MCT-1-expressing cells (lane 6), suggesting that MCT-1 destabilized PTEN via a proteasome pathway. The MCT-1-stimulated AKT phosphorylation was partly suppressed by MG132 treatment, suggesting it was also regulated independently of PTEN. To further answer if MCT-1 decreases PTEN level through an ubiquitin–proteasome pathway, an *in vivo* ubiquitination assay was studied in doxycycline-inducible H1299/TR cell line (p53-null) to  enhance  conditionally MCT-1 expression (Figure 1d). Subsequently, the cells were transiently transfected with the vector encoding HA-ubiquitin and treated with or without MG132, immunoprecipitated (IP) with HA antibody (Ab) and detected by PTEN Ab. We found that more ubiquitinated PTEN was observed in the MCT-1-overexpressing cells than the control set, showing that MCT-1 promotes PTEN degradation via an ubiquitin–proteasome pathway. Moreover, the relative *PTEN* mRNA levels expressed in the MCF-10A cells were examined, we observed that *PTEN* mRNA levels in the ectopic MCT-1-expressing cells were reduced to 46% of that of the control cells (Figure 1e). Therefore, MCT-1 inhibits PTEN gene expression, protein phosphorylation and stability.

To study whether PTEN deficiency enhances the MCT-1 oncogenic effects, MCF-10A cells without (control) or with MCT-1 induction (MCT-1) were transfected with the pMKO.1 short hairpin PTEN (shPTEN) to deplete PTEN protein in both control (control/−PTEN) and MCT-1-inducing cells (MCT-1/−PTEN). After starving for 24 h (-activation), the cells were reactivated with serum for 30 min (+activation) and it was observed that the active AKT (ser473) and EGF receptor (EGFR) (tyr1068) were enhanced in the MCT-1/−PTEN cells with no detectable change in the extracellular signal-regulated kinase (thr202/tyr204) activation (Figure 1f, lane 8). In consistence, under a stringent condition lacking serum and essential growth factors, the survival rate of MCT-1/−PTEN cells were highly induced relative to the other cohorts (control, MCT-1, control/−PTEN) (Figure 1g). The combined effect of PTEN knockdown and MCT-1 induction thus greatly reduced growth factor dependence for survival.

### Overexpressing MCT-1 perturbs the mitotic process in the PTEN-deficient cells

PTEN regulates chromosomal segregation and cytokinesis.^15^ To examine whether MCT-1 further disturbs mitotic progress in the absence of PTEN protection (Supplementary Figure S2a), the MCF-10A cells were arrested at prometaphase by nocodazole treatment for 24 h and then released for 1 h, allowing more cells entering late mitotic stage. The mitotic spindle asters and microtubule structure were detected with NuMA (nuclear-mitotic apparatus) and α-tubulin Abs, respectively; it was observed that the majority of control cells displayed a regular spindle configuration and only a few mitotic cells (4.73%) exhibited a multipolar spindle structure (Supplementary Figure S2b). Conversely, the distorted spindle arrays developed from multipolar regions were more abundantly observed in the MCT-1/−PTEN cells (31.34%) than in the ectopic MCT-1 cells (11.37%) and the control/−PTEN cells (12.14%). In support of mitotic deregulation, the p190B (3.5-fold), NuMA (2.6-fold) and histone H3 phosphorylation (ser10) (11.6-fold) were highly induced in the MCT-1/−PTEN cells compared with the other cohorts (control, MCT-1, control/−PTEN) (Supplementary Figure S2c). Spindle multipolarity increases the incidence of chromosomal miss-segregation and nuclear aberration through the subsequent cell division. Time-lapse microscopy was thus conducted and it was observed that the control/−PTEN cells entered mitosis (0:00) (h:min), rapidly formed a cleavage furrow (0:19) and severed the midbody to complete mitosis (2:20) (Supplementary Figure S2d). Although the MCT-1/−PTEN cells entered mitosis (0:00) and quickly formed a cleavage furrow (0:20), the midbody remained connected (2:50) and two daughter cells still tethered together (4:20). Unexpectedly, the two dividing cells were fused producing a giant binucleated cell (5:50) (Supplementary Figure S2d).

### MCT-1 promotes multinucleation via the Src/p190B signaling amplification

**Figure 2:**
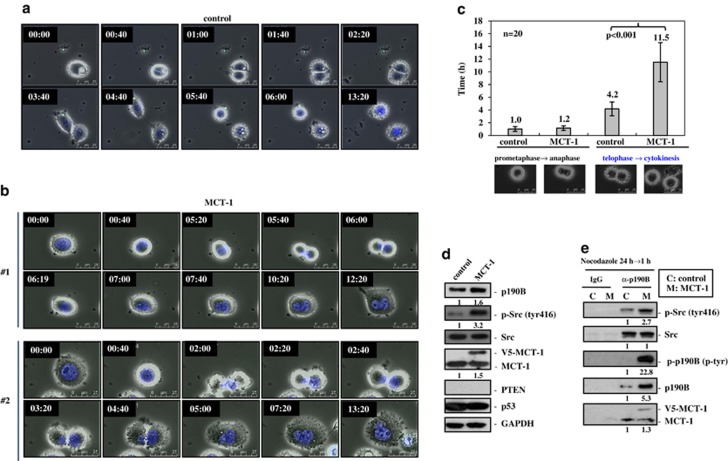
Enhanced MCT-1 expression induces abscission failure and cell–cell fusion. The PTEN-null MDA-MB-468 cancer cells were analyzed. (**a**) Time-lapse microscopy was performed and it revealed that the vector control cells entered metaphase (0:40) and completed the mitotic division at 6:00 (h:min). (**b**) Time-lapse microscopy identified that MCT-1 expression impaired cytokinesis and increased cell–cell fusion, thereby generating giant multinucleated cells. Two typical processes of binucleation were indicated (nos. 1 and 2). (**c**) The time length in each mitotic stage was analyzed. The MCT-1-overexpressing cells spent longer time in late mitosis from telophase to cytokinesis than the control cells. (**d**) The cells were activated by the serum for 30 min after starvation for 24 h. Src phosphorylation and p190B expression were enhanced by the ectopic MCT-1 expression (V5-MCT-1). (**e**) The p190B was IP after the cells treated by nocodazole for 24 h and released for 1 h. The intrinsic/ectopic MCT-1 was co-IP with the active Src. The active phosphorylated p190B and Src were highly induced by MCT-1.

The fluorescence time-lapse microscopy of the mitotic progression was further performed in the PTEN-null MDA-MB-468 cells (Figure 2). We observed that the control cells entered mitosis (0:00), divided completely into two daughter cells (6:00) and with no cytoplasmic fusion occurred during 13 h of observation (Figure 2a). However, MCT-1 expression delayed mitotic progression in that the cell (no .1) entered mitosis (0:40) and formed a cleavage furrow at a later time point (5:40), but the cytoplasmic membrane fusion occurred promptly (6:19) generating a giant binucleated cell (7:40) (Figure 2b). In another case, the MCT-1-expressing cell (no. 2) entered mitosis (0:40) but formed an asymmetric cleavage furrow (2:00) where the midbody between two daughter cells still tethered together producing an abnormal cell with binuclei of unequal sizes (5:00). The average of mitotic time length was assessed and it observed no time difference in early mitosis from prometaphase to anaphase between the control and MCT-1-expressing cells (Figure 2c). The marked divergence was recognized in late mitosis from telophase to cytokinesis, where the ectopic MCT-1 cells took approximately 11.5 h and the control cells spent only 4.2 h, supporting that MCT-1 mainly disturbed the late mitotic stage in PTEN-null background.

Reduced p190RhoGAP is essential for the completion of cytokinesis, which hyperactive p190A and in turn impairs cytokinesis in the PTEN-null cancer cells.^18^ To investigate which p190RhoGAP was deregulated by MCT-1, the *p190A* and *p190B* mRNA levels were analyzed in the MDA-MB-468 breast cancer cells (Supplementary Figure S3). The mRNA level of *p190B* but not *p190A* was increased upon MCT-1 overexpression (a), and conversely, *p190B* mRNA level was decreased after MCT-1 knockdown (b). Along with mitotic perturbation in ectopic MCT-1 cells, the levels of active Src (tyr416) and p190B showed a 3.2-fold increase and a 1.6-fold increase, respectively (Figure 2d). To examine if Src and p190B interact with MCT-1, the MDA-MB-468 cells were nocodazole-arrested at prometaphase stage for 24 h and released for 1 h. Immunoprecipitation study identified not only that p190B protein was enriched but also that the tyrosine-phosphorylated p190B (p-tyr) and the active Src (tyr416) were also greatly enhanced upon MCT-1 overexpression (Figure 2e, lane 4). Intriguingly, p190B bound Src and interacted with the intrinsic and ectopic MCT-1, proposing the close proximity interactions between these proteins at the later mitotic process.

**Figure 3:**
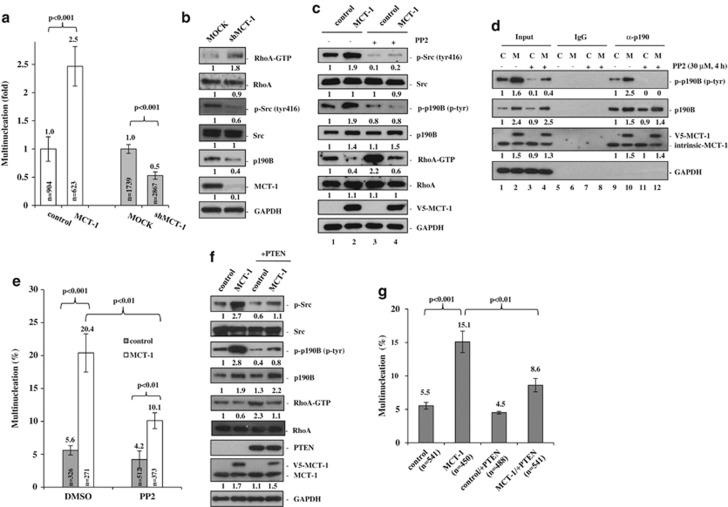
MCT-1 induces multinucleation through the Src signaling pathway. MDA-MB-468 cells were examined. (**a**) The multinuclear effect was compared between the vector control (control), ectopic MCT-1-expressing (MCT-1), non-silencing control (MOCK) and MCT-1 silencing (shMCT-1) cells. Multinuclear frequencies were increased by MCT-1 overexpression but suppressed by MCT-1 knockdown. (**b**) The active phosphorylated RhoA (RhoA-GTP) and Src (tyr416) and p190B amounts were examined before and after MCT-1 depletion. Unlike RhoA activation, the levels of p-Src and p190B were repressed by MCT-1 knockdown. (**c**) Cells were reactivated with serum for 30 min after starvation for 24 h and treatment with an Src inhibitor (PP2) for 4 h. PP2 suppressed the active Src (tyr416) and p190B (p-tyr) but increased RhoA-GTP in the ectopic MCT-1 cells. (**d**) Immunoprecipitation of p190B was conducted and PP2 decreased the active p190B (p-tyr) (lanes 11 and 12) but not the p190B/MCT-1 interaction (lane 12). (**e**) PP2 attenuated the multinuclearity promoted by MCT-1. (**f**) The cells with or without PTEN restoration were analyzed after starving for 24 h and reactivating with serum for 30 min. In contrast to the reduced phosphorylation of Src (tyr416) and p190B (p-tyr), the RhoA-GTP was increased in the ectopic MCT-1 cells expressing PTEN (MCT-1/+PTEN). (**g**) Multinuclear frequencies and numbers of cells scored in each group were indicated. Re-expression of PTEN inhibited the multinucleation induced by MCT-1.

To investigate whether enhanced MCT-1 activation is the main reason for the nucleation aberration, the microscopic evaluation was conducted and it was found that there was a 2.5-fold increase in multinuclear MCT-1-expressing cells and frequently with more than two nuclei (Figure 3a). However, knockdown of MCT-1 (shMCT-1) reduced the multinucleated effect by half and most of the control cells were mononuclear. Accompanied with reduced multinucleation upon MCT-1 depletion, the RhoA activation was increased when Src activation (phosphorylation of tyr416) and p190B expression were reduced markedly (Figure 3b). MCT-1 likely augments the Src/p190B signaling cascade, which inhibits RhoA activation (RhoA-GTP) and mediates multinucleation. To answer if Src mediates p190B/RhoA signaling activation in the context of MCT-1 induction, an Src inhibitor (PP2) was treated with the MDA-MB-468 cells. We found that PP2 suppressed the active Src (try416) and p190B (p-tyr) and, as expected, increased RhoA activation (RhoA-GTP) in contrast to ectopic MCT-1 expression (Figure 3c, lanes 2 vs 4). The results suggest that MCT-1 works through Src to activate p190B and to inhibit RhoA. To answer if Src activity determines the interaction between MCT-1 and p190B, immunoprecipitation assay was performed and it identified that p190B still interacted with MCT-1 despite the Src/p190B signaling inhibited by PP2 (Figure 3d, lane 12). Under such a condition, multinuclearity was reduced by half in the ectopic MCT-1 cells (20.4% vs 10.1%); however, PP2 had no substantial influence on the control cells (5.6% vs 4.2%) (Figure 3e). Therefore, MCT-1/p190B interaction is independent of Src activation; however, the interaction may still have an important role in promoting multinucleation.

We next investigated the necessity of PTEN for protection against multinucleation, the MDA-MB-468 cells were reintroduced into the *PTEN* gene. Significantly, re-expressing PTEN reduced active phosphorylation of Src and p190B but increased the active RhoA-GTP level in the ectopic MCT-1 cells (Figure 3f, lane 4). Consistently, we observed a lower incidence of multinucleation through gain-of-function PTEN in MCT-1-expressing cells (MCT-1/+PTEN) than the relative control cells (control/+PTEN) (Figure 3g). Concurrent with significant activation of Src (tyr416) and p190B (p-tyr) in the MCF-10A cells (Supplementary Figure S4a, lane 4), the active RhoA-GTP level was also reduced markedly in the MCT-1/−PTEN condition, indicating that Src/p190B signaling was synergistically promoted by MCT-1 expression and PTEN deficiency. Through abortive cytokinesis and cytoplasmic membrane fusion following cell plate formation (Supplementary Figure S2d), we noticed a high rate of multinucleation in MCT-1/−PTEN cells comparative to the other groups (control, MCT-1, control/−PTEN) (Supplementary Figure S4b). These data confirm that MCT-1 expression and PTEN loss cooperatively interfere with mitotic completion, but PTEN restoration suppresses the MCT-1-induced multinucleation.

**Figure 4:**
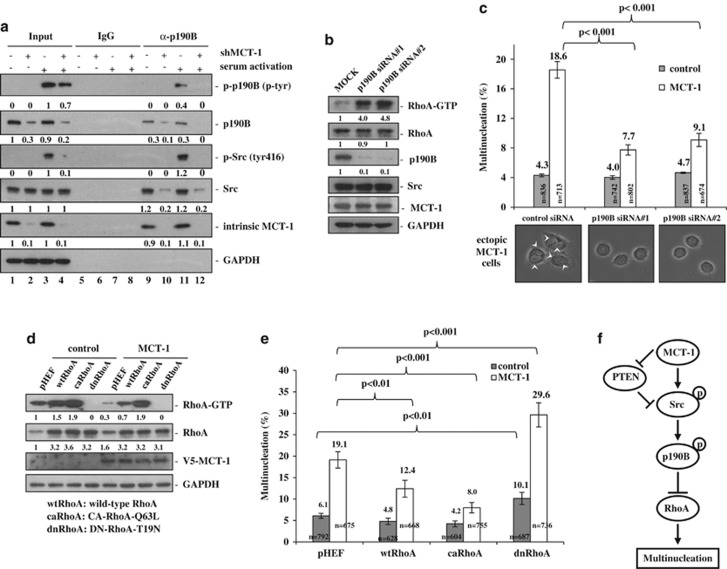
MCT-1 promotes multinucleation dependent on the p190B/RhoA function. MDA-MB-468 cells were analyzed. (**a**) Cells were reactivated for 30 min after starving for 24 h. Immunoprecipitation of p190B was conducted and was it observed that MCT-1 knockdown (shMCT-1) reduced the signal activation and interaction of Src/p190B. (**b**) RhoA activation and multinucleation were analyzed after depleting p190B by  shRNA (nos. 1 and 2). (**c**) Knockdown of p190B activated RhoA but suppressed the MCT-1-induced multinuclearity. (**d**) The vectors encoding wtRhoA, caRhoA and dnRhoA were transfected to modify RhoA activity. (**e**) MCT-1-induced multinucleation was prevented by caRhoA and wtRhoA but promoted by dnRhoA. (**f**) Overexpression of MCT-1 inhibits PTEN function and works through the Src/p190B/RhoA signaling cascade to induce multinucleation.

To evaluate if MCT-1 mediates the Src/p190B interaction and signaling activation, MDA-MB-468 cells were starved and reactivated with serum followed by p190B immunoprecipitation (Figure 4a). We found that the levels of total Src and active Src (tyr416) associated with p190B were increased markedly only when MCT-1 was present (lanes 9 and 11), and such interaction was much decreased through MCT-1 depletion (shMCT-1) (lanes 10 and 12). Knockdown of MCT-1 abolished the Src-p190B activation and suppressed the interaction of active Src and p190B, suggesting that the MCT-1-enhanced interaction facilitated Src activation of p190B. The role of p190B in multinucleation was next assessed by the interference of *p190B* gene expression (p190B siRNA nos. 1 and 2) (Figure 4b). Unlike RhoA activation, multinuclear frequencies were significantly reduced upon p190B depletion in the MCT-1-expressing cells (Figure 4c). We further elucidated if RhoA activity was indeed implicated in the multinucleated effect; the MDA-MB-468 cells were introduced with wild-type RhoA (wtRhoA), constitutive-active RhoA (caRhoA) or dominant-negative RhoA (dnRhoA) (Figure 4d). We found that RhoA activation (RhoA-GTP) was completely suppressed by dnRhoA, but it was enhanced by wtRhoA and greater promoted by caRhoA. Significantly, both wtRhoA and caRhoA prevented the MCT-1-induced multinucleation, whereas dnRhoA advanced multinuclearity (Figure 4e). Putting together, we have identified a novel mechanism of multinuclearity under MCT-1 oncogenic stress via the Src/p190B/RhoA signaling cascade in the PTEN-null background (Figure 4f).

### Targeting MCT-1 reduces chromosomal polyploidy and tumor growth of the PTEN-null breast cancer cells

**Figure 5:**
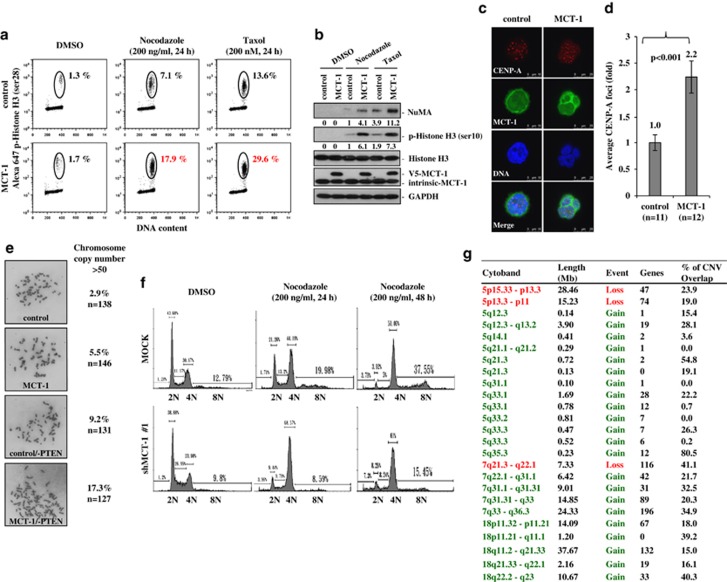
MCT-1 status affects mitotic progression and chromosome stability. MDA-MB-468 (**a**–**d** and **g**) and MCF-10A (**e** and **f**) cells were analyzed. (**a**) Upon treatment with nocodazole and taxol, mitotic populations were examined by flow cytometry after immunostaining the phosphorylated histone H3 (ser28). (**b**) The levels of NuMA and phospho-histone H3 (ser10) expression were examined upon nocodazole and taxol treatment. (**c**, **d**) CENP-A foci were evaluated in the multinuclear interphase cells, and they were relatively increased through MCT-1 induction. (**e**) Mitotic spread studies identified more amplified chromosome copy number (>50) upon MCT-1 overexpression and PTEN loss (MCT-1/−PTEN). (**f**) Cell cycle profiling and polyploidy populations were analyzed by flow cytometry after nocodazole treatment. MCT-1 knockdown (shMCT-1) suppressed the polyploidization. (**g**) Array-based comparative genomic hybridization (array CGH) study identified the exact locations of the chromosome loss (red) and gain (green) at chromosomes 5, 7 and 18 regions due to MCT-1 overexpression. The ratios of chromosome copy number variation (percentage of copy number variation (%CNV)) are indicated.

Mitotic checkpoint ensures the accuracy of cell division. Using flow cytometry analysis, we observed that 59.2% of the MDA-MB-468 cells were arrested at G2/M transition upon nocodazole treatment for 24 h (Supplementary Figure S5a). However, the MCT-1-overexpressing cells were less responsive to the G2/M arrest and, therefore, up to 44% of the cells were still retained at G1 stage. Similar effect was noticed in the MCT-1-overexpressing cells with taxol treatment for 24 h where fewer cells (47.7%) were arrested at G2/M stage compared with that of the control cells (60.6%). The decreased G2/G1 ratio in the ectopic MCT-1 cells suggested that MCT-1 perturbs the G2/M checkpoint in the PTEN-null context (Supplementary Figure S5b). Furthermore, to  identify specifically the cell populations with chromosome condensation during mitosis, the flow cytometry results confirmed that more phospho-histone H3 (ser28)-positive cells were detected because of MCT-1 induction (17.9% and 29.6%) after microtubule disruption by nocodazole and taxol, respectively (Figure 5a). In support of mitotic promotion following the treatment of microtubule toxins, we observed that the mitotic markers, NuMA and the phospho-histone H3 (ser10), were highly induced by MCT-1 overexpressing (Figure 5b), confirming that MCT-1 enhances the mitotic progression.

Mitotic abnormality induces polyploidization. CENP-A is a centromeric protein required for kinetochore assembly and stability.^32^ Supernumerary CENP-A foci correspond to amplification of chromosomal copy number in the cell. The numbers of CENP-A foci (red) (upper panel) were examined at interphase stage by immunofluorescence microscopy (Figure 5c). Compared with the control MDA-MB-468 cells, the average CENP-A foci showed a 2.2-fold increase in the multinucleated MCT-1-overexpressing cells (Figure 5d). The amplification of CENP-A foci rationally explains high incidence of cytokinesis failure causing multinucleation and polyploidization under the MCT-1 oncogenic stress. Consistently, the data of mitotic chromosome spread identified a higher percentage of the MCT-1/−PTEN cells (17.3%) with amplified chromosome copy number (>50) than the other MCF-10A cellular contexts (control, MCT-1, control/−PTEN) (Figure 5e), supporting that MCT-1 promotes chromosome abnormalities in the absence of PTEN protection.

Furthermore, we assessed if MCT-1 depletion can renovate the G2/M and polyploidy checkpoint control in the MDA-MB-468 cells (Figure 5f). The flow cytometry data showed that 12.79% of the MOCK control cells contained intrinsic polyploidization with DNA content of more than 4*N*. Polyploidy was accumulated progressively when the cells were continually exposed to nocodazole for 24 h (19.98%) to 48 h (37.55%). Surprisingly, the polyploidy populations were reduced by half upon MCT-1 depletion (shMCT-1 no. 1). A larger number of the MCT-1 knockdown cells were arrested at the G2/M phase (68.57%) than the MOCK control cells (44.19%) after microtubule damage for 24 h. Therefore, targeting MCT-1 can prevent the PTEN-null cancer cells from bypassing the polyploidy and G2/M checkpoints.

Array-based comparative genomic hybridization was again investigated chromosome copy number variation (CNV) caused by MCT-1 in a genome-wide screening (Figure 5g and Supplementary Figure S6). The Cy3-labeled DNA probes from MCT-1-overexpressing cells and Cy5-labeled DNA probes from control MCF-10A cells were simultaneously hybridized with the chromosome array to identify whether the chromosomal aberrations occurred specifically via overexpressing MCT-1 that inhibited PTEN expression. The copy number changed in any region within a chromosome is indicated by a segmentation chart along the chromosome regions and by the log_2_ ratio of fluorescent signals obtained from the two scanning channels. A ratio of +0.2 or −0.2 is set as a cutoff value for defining the significant variations. Under these criteria, we observed the aberrations on chromosomes 5, 7 and 18 (Supplementary Figure S6). The segments of chromosome amplification are highlighted in green, and the regions of chromosome loss are underlined in red on the cytoband region of chromosomes 5 (Supplementary Figure S6a), 7 (Supplementary Figure S6b) and 18 (Supplementary Figure S6c). Detailed information for chromosome gain or loss, the number of genes located in these regions and the percentage of copy number variations are listed in Figure 5g. The incidences of amplifications (marked in green) were identified in the chromosome 5q region, in the chromosome 7q21.3–q36.3 regions and in a large segment of chromosome 18 covered from 18p11.32 to 18q23 region. Moreover, the results of chromosome deletions (marked in red) were identified at regions of 5p15.33–p13.3, 5p13.3–p11 and 7q21.3–q22.1. The genes located on these mutated chromosomes may contribute to the MCT-1 oncogenic potential.

**Figure 6:**
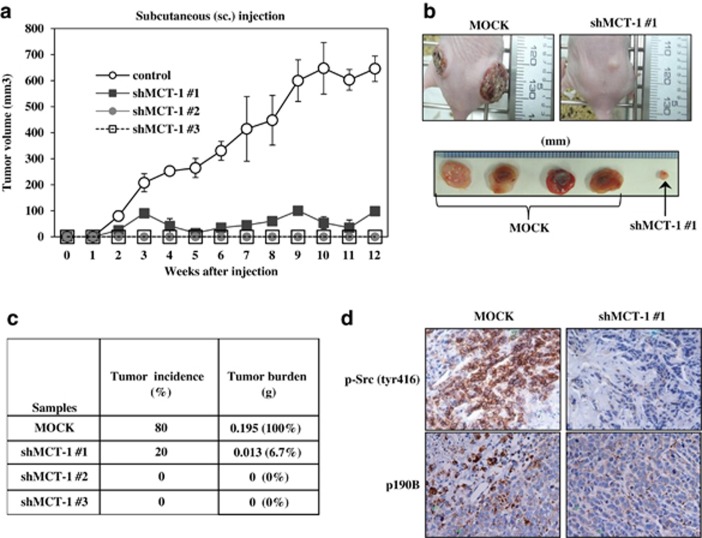
Knockdown of MCT-1 inhibits tumorigenicity. (**a**) MDA-MB-468 cells were subcutaneously (s.c.) injected into the nude mice. Tumor volumes were measured weekly. (**b**) Mice were killed at the end point of 13 weeks. Tumor burdens were reduced markedly upon MCT-1 knockdown (shMCT-1 no. 1) compared with the non-silence control cells (MOCK). (**c**) Tumor incidences and burdens were analyzed. MCT-1 depletion (shMCT-1 nos. 1, 2 and 3) significantly inhibited tumor growth. (**d**) Immunohistochemical study detected low levels of p190B and active Src (tyr416) in the tumors emerged from the MCT-1 knockdown cells.

To assess the tumorigenic role of MCT-1, the MDA-MB-468 cells without (MOCK) or with MCT-1 knockdown (shMCT-1 nos. 1, 2 and 3) were subcutaneously injected into the BALB/c nude mice (*n*=6). Tumor development was monitored weekly and an obvious difference in tumor volume was recognized after 4 weeks (Figure 6a). At the end point of 13 weeks, we observed a significant difference in tumor masses between MCT-1 knockdown and MOCK sample (Figure 6b). The tumor incidence dropped to 20% owing to MCT-1 knockdown (shMCT-1 no. 1) and no significant tumor development in two other cohorts (shMCT-1 nos. 2 and 3) (Figure 6c). Tumor burdens of the xenograft cancer cells with MCT-1 reduction (shMCT-1 no. 1) were also markedly reduced to 6.7% of that identified in the control xenografts (MOCK). Immunohistochemistry analysis confirmed that MCT-1 deficiency suppressed the expression levels of p190B and active Src (tyr416) in the tumors (Figure 6d). Accordingly, reduced MCT-1 activity in the PTEN-null cancer cells substantially abolishes tumorigenic growth and inhibits the Src/p190B signaling activation *in vivo*.

### Relationship of MCT-1 with PTEN and p190B expression in human breast cancer

**Figure 7:**
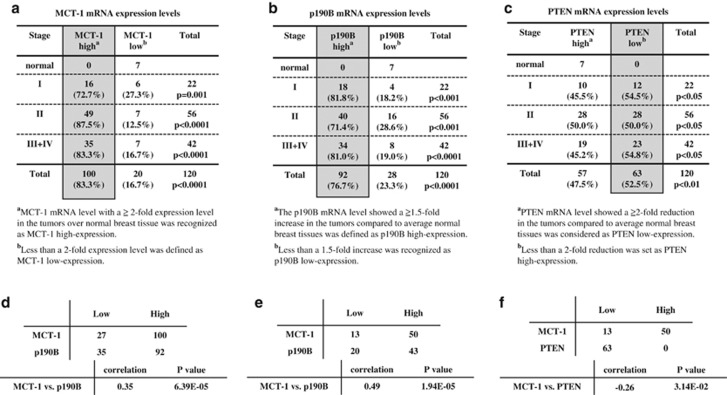
Clinical relevance of MCT-1, p190B and PTEN expression in human breast cancers. TissueScan breast cancer tissue cDNA arrays (BCRT I, III and IV) were analyzed by quantitative real-time polymerase chain reaction. (**a**) Relative MCT-1 expression levels in human breast cancers were studied. The *MCT-1* mRNA level identified in each tumor sample was normalized to β*-actin* mRNA and calibrated to the overall mean of *MCT-1* mRNA level in normal breast tissues. (**b**) Relative p190B expression levels in human breast cancers were studied. The *p190B* mRNA level detected in each tumor biopsy was normalized to *β-actin* mRNA and compared with the mean of *p190B* mRNA level in normal breast tissues. (**c**) Relative PTEN expression levels in human breast cancers were analyzed. The *PTEN* mRNA level identified in each tumor sample was normalized to *β-actin* mRNA and calibrated to the overall mean of *PTEN* mRNA level in normal breast tissues. The comparison between normal breast tissues and different stages of breast tumors were analyzed by the *X*^2^ test (**a**–**c**). A *P*-value of <0.05 is considered to be statistically significant. (**d**) The correlation between the expression of MCT-1 and p190B was evaluated. If and only if MCT-1 or p190B expression in each sample is larger than the expression in any of the normal tissues is defined as ‘high'. Based on this definition of ‘high' and ‘low' expression, the dichotomized data for the 127 samples, MCT-1 and p190B expression are positively correlated (*P*-value 6.39 × 10^−5^). (**e**) The PTEN expression levels in breast tumors showing a twofold lower than average of the normal breast tissues were analyzed (*n*=63). A significant positive correlation between the MCT-1 and p190B expression was identified (*P*-value 1.94 × 10^−5^). (**f**) A negative correlation between the MCT-1 and PTEN expression was identified in the PTEN-low tumors (*n*=63) (*P*-value 3.14 × 10^−^^2^). The Pearson's correlation coefficient is used to measure the relationship between two indicated genes (**d**–**f**).

The clinical relevance of *MCT-1* gene activation in relation with *PTEN* and *p190B* gene expression was studied using the TissueScan breast cancer tissue qPCR array (OriGene Technologies, Inc., Rockville, MD, USA). *MCT-1* mRNA expressed in different stages of human breast carcinomas (*n*=120) and normal breast tissues (*n*=7) were analyzed. A twofold increase over the mean of *MCT-1* mRNA level in normal breast tissues were recognized as MCT-1 high expression in the cancer. In this criteria, the expression of *MCT-1* mRNA was induced in stage I (72.7% *P*=0.001), stage II (87.5% *P*<0.0001) and stages III–IV (83.3% *P*<0.0001) of the cancer patients (Figure 7a). Overall, 83.3% of the breast cancer patients showed increase in *MCT-1* mRNA level compared with the normal tissues (*P*<0.0001), revealing MCT-1 overexpression in most breast cancers. MCT-1 expression induces *p190B* gene activation (Supplementary Figure S3a). Because *p190B* mRNA levels in normal breast tissues were relatively higher than that of *MCT-1* gene, therefore, we defined p190B upregulation as its expression with a 1.5-fold increase over that of average normal breast tissues. Accordingly, *p190B* gene activation was observed in stage I (81.8% *P*<0.001), stage II (71.4% *P*<0.001) and stages III–IV (81% *P*<0.0001) of breast cancers (Figure 7b). Of the 120 cancer samples, there were 76.7% of them showed *p190B* gene stimulation relative to normal breast tissues (*P*<0.0001). Moreover, *PTEN* mRNA levels in these tumor biopsies were also studied (Figure 7c). As compared with the mean of *PTEN* mRNA expression in normal breast tissues, the *PTEN* mRNA levels with a twofold reduction in different stages of human breast carcinomas were defined as PTEN low expression. Under such a criteria, PTEN reduction was observed in stage I (54.5% *P*<0.05), stage II (50% *P*<0.05) and stages III–IV (54.8% *P*<0.05) of the patients. Overall, 52.5% of the tumor cases exhibited a PTEN suppression compared with normal breast tissues (*P*<0.01).

To study the clinical relationship of MCT-1 with p190B and PTEN expression, we used the expression levels in normal tissues to define the threshold. For a given gene, we dichotomize its expression level in each sample into ‘low' and ‘high', respectively; it is ‘high' if and only if its level is larger than the expression in any of the normal tissues. Figure 7d shows the dichotomized data for the 127 samples, from which the expression levels of MCT-1 and p190B are significantly and positively correlated with Pearson's correlation of 0.35 and *P*-value 6.39 × 10^−5^. Similar results are observed in the tumors (*n*=63) with twofold PTEN expression lower than that of the normal breast tissues (Figure 7e), from which we also identify a significant positive correlation between the expression levels of MCT-1 and p190B, with Pearson's correlation of 0.49 and *P*-value of 1.94 × 10^−5^. As assessed further, the association between MCT-1 and PTEN among the samples having low-level PTEN (Figure 7f), MCT-1 and PTEN are negatively correlated with Pearson's correlation of −0.26 and *P*-value 3.14 × 10^−2^. Taken together, MCT-1 is induced abundantly in human breast cancers with p190B stimulation and with PTEN suppression.

## Discussion

PI3K/AKT signaling pathway regulates cell growth, proliferation and survival.^33^ Hyperactive PI3K/AKT has been identified in breast, ovarian and many other cancers.^34, 35, 36^ Deletion or mutation of the *PTEN* gene and under the oncogenic stress, which highly augments the PI3K/AKT activation, contribute to neoplastic transformation or metastatic potential in a wide spectrum of human cancers.^37, 38, 39^ MCT-1 prevents PTEN function through a decrease in PTEN protein stability and gene activation (Figure 1). MCT-1 stimulates both Src and PI3K pathways (Supplementary Figures S1a and c). In the context of PTEN deficiency or ablation (Figures 1f and 3f), MCT-1 overexpression further stimulates the activation of AKT, EGFR and Src, thereby enhancing cell survival.

Genetic mutations increase tumor predisposition. Defects in chromosomal segregation, cell cycle checkpoint and DNA damage repair cannot protect against the reproduction of abnormal cells with genomic aberrations that potentially induce the malignant progression.^40, 41, 42^ The activation of oncogene contributes to tumor development not only by inducing proliferation but also by destabilizing genomic structure and reprogramming stem cells.^43^ For example, c-Myc-overexpressing cells progress to intraepithelial neoplasia and adenocarcinoma lesions with marked heterogeneity in loss of *PTEN* and *p53* genes.^44^ Loss of p53 combined with c-Myc overexpression in astrocytes induces the expression of stem cell makers, which also promotes glioma pathogenesis.^45^ Similarly, MCT-1 oncogenic activation may have selective pressure to trigger the loss of tumor suppressor such as PTEN and p53, and thus promoting tumorigenesis. PTEN physically and genetically interacts with p53, the guardian of genome structure.^5^ Losses of PTEN and p53 cooperatively converge on c-Myc to enhance cell proliferation, self-renewal and tumorigenic potential.^46^ We now demonstrate that MCT-1 promotes PTEN degradation through an ubiquitin–proteasome pathway in a p53-independent manner, and also discover a novel oncogenic role of MCT-1 in enhancing mitotic catastrophe, checkpoint failure and neoplastic multinucleation, leading to genomic aberrations particularly in PTEN-deficient context. It is still unknown whether MCT-1 and Myc oncogenes have crosstalk in the tumorigenic process.

MCT-1 expression and PTEN loss synergistically promote cytokinesis failure and multinucleation (Supplementary Figures S2d and S4b). Likewise, enhanced MCT-1 activation in the p53-deficient cells impairs mitotic development, G2/M checkpoint and genomic stability upon microtubule disruption or DNA damage.^27,28^ Genetic mutations are thus accumulated progressively as a consequence of the MCT-1 oncogenic activation and the tumor suppressor dysfunction. Following spindle multipolarity and cytokinesis failure, cell–cell fusion is induced by expressing MCT-1 in the PTEN-deficient cells (Supplementary Figures S2 and S4b). Catastrophic mitosis is also promoted significantly while increasing MCT-1 in the PTEN-null cancer cells (Figure 2b). Targeting MCT-1 improves the mitotic regulation and prevents polyploidization in the PTEN-null cancer cells (Figure 5f), suggesting that the mitotic progression and polyploidy checkpoint may work most efficiently when PTEN and MCT-1 are in functional balance.

The tyrosine-1109 residue of p190B is homologous with the tyrosine-1105 residue on p190A that is phosphorylated and activated by Src.^47^ We speculate that MCT-1 enhances p190B activity possibly through Src phosphorylation of p190B at the tyrosine-1109. The enhanced tyrosine phosphorylation of p190B regulates chromosomal segregation in the PTEN-null cancer cells.^20^ The hyperactive p190A perturbs cytokinesis.^19^ Similarly, the highly activating p190B by MCT-1 may cause disproportional contraction of the mitotic furrow site, resulting in asymmetrical cell division, chromosomal miss-segregation and cytokinesis failure. Resembling the dynamic distribution of p190B during mitosis,^20^ MCT-1 is temporarily located at centrosome and midbody,^28^ where MCT-1 may encounter and cooperate with p190B to regulate mitotic progression. The Src activity-independent p190B/MCT-1 interaction (Figure 3d) and a direct involvement of MCT-1 in the signaling activation of Src/p190B (Figures 3b and 4a) imply the proximity complex of MCT-1/p190B/Src facilitating Src phosphorylation and activation of p190B. Reduced MCT-1 activity effectively suppresses Src/p190B signaling to prevent neoplastic multinucleation in the PTEN-null cancer cells, showing an underlying mechanism of multinucleation induced by the oncogene.

PTEN activity inhibits cell invasion and migration.^48^ The ectopic MCT-1 expression increases the functionally inactive PTEN (ser380) (Supplementary Figure S1a). PTEN may antagonize MCT-1 function in the Src/p190B signaling pathway, thereby ensuring proper mitotic division and maintaining the integral nuclear/chromosomal structure. More detailed investigation is necessary to fully understand whether MCT-1 overexpression alongside PTEN loss indeed promotes the Src/p190B signaling in the development of tumor. Results from analysis of clinical specimens offer that *MCT-1* gene activation may be recognized as a novel biomarker for early diagnosis of breast tumor development. Significant correlation between MCT-1 overexpression with PTEN suppression and p190B induction in human breast cancers is firstly demonstrated (Figure 7). *MCT-1* and *p190B* genes are concurrently stimulated, supporting their collaborations in the development of mammary tumor. The xenograft tumor studies have indicated that targeting MCT-1 decreases the active phosphorylated Src and p190B *in vivo* (Figure 6d), suggesting that MCT-1 controls cancer cell propagation and tumor progression via Src/p190B signaling amplification.

Src activates AKT via inhibition of PTEN in breast cancer.^24^ Herceptin is a chemotherapeutic agent for HER2-positive metastatic breast cancer.^49^ The herceptin-resistant breast cancers are often identified with Src activation because the PTEN loss cannot dephosphorylate and inactivate Src.^25^ Src pathway is, therefore, recognized as an ideal therapeutic target for administration of breast cancer with PTEN mutation. MCT-1 facilitates Src activation; therefore, elucidating their molecular interaction will help identify new and effective therapeutic strategies for cancer(s) with Src hyperactivation and/or PTEN deficiency.

## Materials and methods

### Antibodies

See Supplementary Materials.

### Knockdown of PTEN and modification of MCT-1

MDA-MB-468, MDA-MB-231 and MCF-7 cells were virally transfected with pLXSN or pLXSN/MCT-1 (V5-tagged) as  described previously.^29^ The stable transfectants (control and MCT-1) were cultured in RPMI 1640 medium with 10% fetal bovine serum, 100 U/ml penicillin, 100 μg/ml streptomycin and 350 μg/ml G418. MCF-10A cells transfected with pLXSN/MCT-1 or pLXSN vector were subsequently introduced pMKO.1 or pMKO.1  shPTEN shRNA (Addgene, Cambridge, MA, USA) using Lipofectamine 2000 (Invitrogen, Grand Island, NY, USA). Cells were subcultured in Dulbecco's modified Eagle's medium/F12 complete medium supplemented with 0.5 μg/ml puromycin for 2 weeks. All the transfectants were maintained in Dulbecco's modified Eagle's medium/F12 medium containing 5% horse serum, 100 U/ml penicillin, 100 μg/ml streptomycin, 20 ng/ml EGF, 0.5 μg/ml hydrocortisone, 10 μg/ml insulin, 100 ng/ml cholera toxin and the selection antibiotics (100 μg/ml G418 and 0.5 μg/ml puromycin). The knockdown of MCT-1 using SureSilencing pGeneClip MCT-1  shRNA and MOCK  shRNA plasmids (SuperArray Biosciences Corporation, Valencia, CA, USA) were stably transfected into MDA-MB-468 cells and cultured with 0.5 μg/ml puromycin.

### *In vivo* ubiquitination assay

The H1299TR/control- and H1299TR/MCT-1-inducible cell lines were transiently transfected with pCR3.1-HA-ubiquitin expression plasmid (Addgene) for 24 h. The ectopic MCT-1 cells were induced by 1 μg/ml doxycycline for 12 h, treated by 50 μM MG132 for 12 h and then extracted by RIPA buffer. One microgram of cell extracts was IP with anti-HA Ab (Roche Diagnostics Corporation, Indianapolis, IN, USA) as described. The polyubiquitinated PTEN was detected with PTEN Ab (Cell Signaling, Danvers, MA, USA).

### Inhibition of protein kinase and proteasome activity

See Supplementary Materials.

### Quantitative real-time polymerase chain reaction

Total RNA was extracted using the Trizol reagent (Invitrogen). Two micrograms of total RNA were digested with DNase I and synthesized cDNA using oligo(dT)_12__–18_ primer and SuperScript II reverse transcriptase (Invitrogen). The specific probes for *MCT-1* (human *MCT-1*, Hs00273837_m1), *p190B* (human *ARHGAP5*, Hs00869394_s1, Hs00750732_s1) and *PTEN* (human *PTEN*, Hs00829813_s1) genes were purchased from Applied Biosystems (Grand Island, NY, USA). Quantitative real-time polymerase chain reaction was performed as described previously.^50^

TissueScan qPCR array panel I (BCRT101), III (BCRT103) and IV (BCRT104) (OriGene Technologies, Inc.) were analyzed the mRNA level for each target gene in human breast cancer using quantitative real-time polymerase chain reaction. The clinical information of each sample was retrieved from OriGene (http://www.origene.com/qPCR/Tissue-qPCR-Arrays.aspx). The pathological significances between tumor stages and gene expression were analyzed.

### MTT  assay

See Supplementary Materials.

### Immunofluorescence and fluorescence time-lapse microscopy

See Supplementary Materials.

### Immunoblotting analysis

See Supplementary Materials.

### Immunoprecipitation assay

See Supplementary Materials.

### Flow cytometry analysis

See Supplementary Materials.

### RhoA activity assay

See Supplementary Materials.

### Multinucleation analysis

MDA-MB-468 cells were seeded on 6-well plates at 50% confluence and cultured for 18–24 h. The control and p190B siRNAs (sip190B no. 1: 5′-GCUGAUACAACCACAAUUA-3′ and sip190B no. 2: 5′-GGAAUCAGUUAAACACAAU-3′) were purchased (Thermo Scientific Dharmacon, Pittsburgh, PA, USA). Cells were transfected with the scramble control siRNA or the p190B siRNA using the Lipofectamine RNAiMAX reagent (Invitrogen). The siRNA/reagent complexes were incubated with cells for 4 h before incubating in fresh medium for 3 days.

The pHEF wtRhoA plasmid was used as a template to generate the caRhoA (pHEF caRhoA) according to the manufacturer's protocol of the QuickChange Site-Directed Mutagenesis Kit (Stratagene, La Jolla, CA, USA).^51^ To construct the dnRhoA, the primers used for the site-directed mutagenesis were shown as follows: RhoAQ63L forward (5′-GTGGGACACAGCTGGGCTGGAAGATTATGATCGC-3′) and RhoAQ63L reverse (5′-GCGATCATAATCTTCCAGCCCAGCTGTGTCCCAC-3′). The cells were fixed with 70% methanol for 15 min, stained with 4′,6-diamidino-2-phenylindole (100 ng/ml) and quantified the multinucleated populations via fluorescence microscopy.

### Immunofluorescent detection of phosphorylated histone H3

See Supplementary Materials.

### Cytogenetic study

See Supplementary Materials.

### Array comparative genomic hybridization study and data analysis

See Supplementary Materials.

### Xenograft tumorigenicity and immunohistochemistry study

The 8-week-old female nude mice (BALB/cAnN.Cg-Foxnl^nu^/CrlNarl) were subcutaneously injected with MDA-MB-468 cells (1.3 × 10^6^). Tumor growth was analyzed weekly with caliper measurements. Tumor volumes were calculated by the formula: tumor volume=1/2 (length × width^2^). After cultivation for 13 weeks, the mice were killed to examine tumor incidences and burdens. Immunohistochemistry study of the tumors were performed as described previously.^50^
