# Supplementary material for: MCT-1 expression and PTEN deficiency synergistically promote neoplastic multinucleation through the Src/p190B signaling activation
Source: Oncogene. 2014 May 26;33(43):5109–20. doi: 10.1038/onc.2014.125 (PMC4287651; doi:10.1038/onc.2014.125)
Supplement: Supplementary Figures [file onc2014125x1.pdf]

Supplementary Figures

Figure S1. MCT-1 expression enhances the PI3K/AKT signaling. (**a**) MCF-10A cells starved in serum-free medium for 24 h were treated with LY294002 (PI3K inhibitor) for 4 h before serum activation for 30 min. LY suppressed the MCT-1-enhanced AKT phosphorylation (ser473). The increased PTEN phosphorylation (ser380) suggested that MCT-1 inhibited PTEN activity (lanes 5 and 6). (**b**) MCF-7 cells starved for 24 h were incubated with wortmannin for 4 h and then activated by serum/insulin for 30 min. Wortmannin effectively suppressed MCT-1-stimulated AKT phosphorylation (ser473). (**c**) MCF-10A cells starved for 24 h were then treated with LY, PP2 or UO for 4 h before activation with EGF (+EGF) or serum for 30 min. The MCT-1-induced AKT phosphorylation was well inhibited by LY and partly suppressed by PP2 but not by a MEK inhibitor, UO. (**d**) The MDA-MB-231 cells were cultured with 200 μM cycloheximide for the indicated time intervals. The residual PTEN protein was examined after block of protein biosynthesis. (**e**) PTEN degradation rate and PTEN half-life were evaluated in MDA-MB-468 cells with or without increasing MCT-1 expression.

Fig. S1

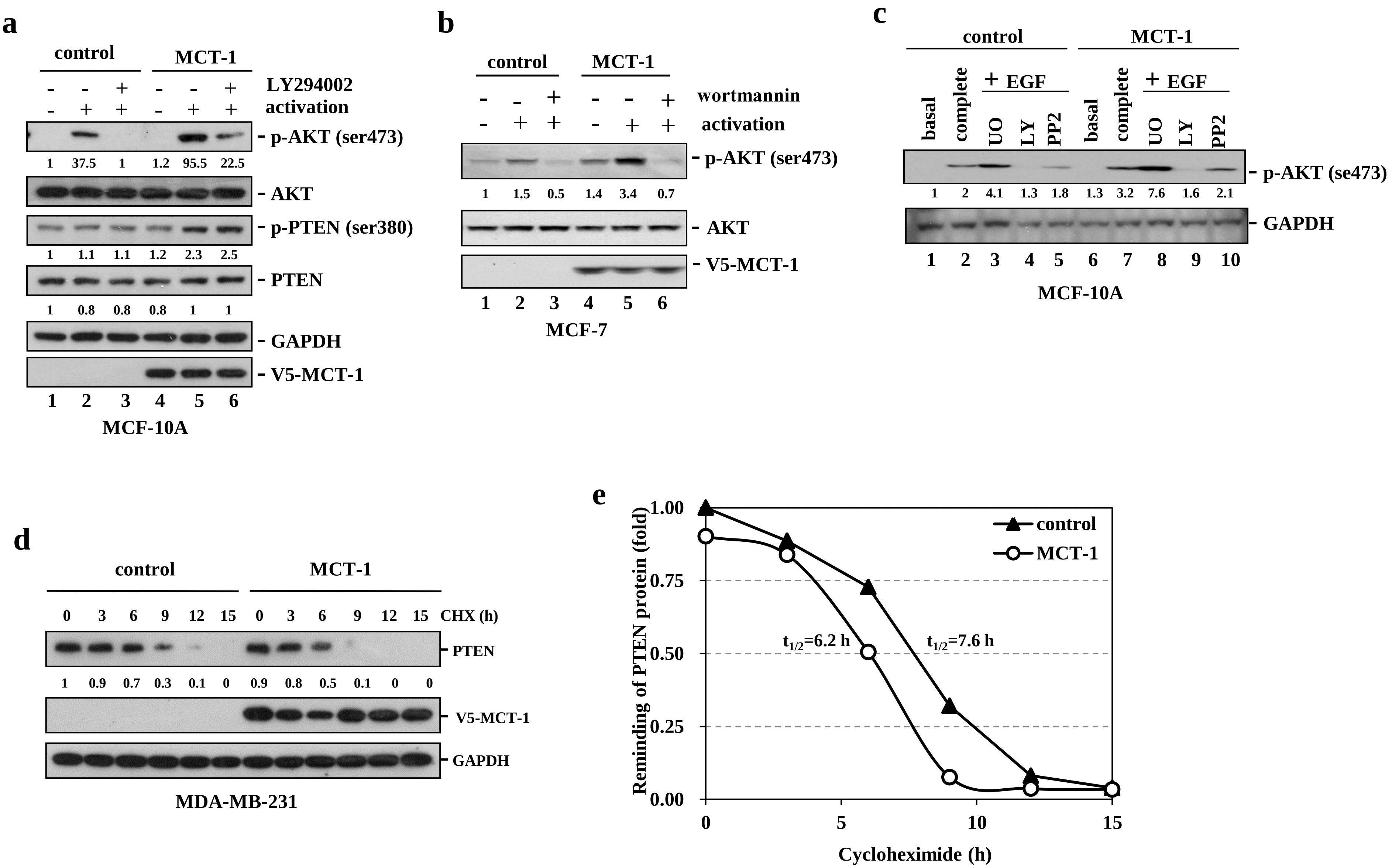

a
control
MCT-1
LY294002
activation
p-AKT (ser473)
1 37.5 1 1.2 95.5 22.5
AKT
p-PTEN (ser380)
1 1.1 1.1 1.2 2.3 2.5
PTEN
1 0.8 0.8 0.8 1 1
GAPDH
V5-MCT-1
1 2 3 4 5 6
MCF-10A
b
control
MCT-1
wortmannin
activation
p-AKT (ser473)
1 1.5 0.5 1.4 3.4 0.7
AKT
V5-MCT-1
1 2 3 4 5 6
MCF-7
c
control
MCT-1
basal
complete
+ EGF
UO
LY
PP2
p-AKT (se473)
1 2 4.1 1.3 1.8 1.3 3.2 7.6 1.6 2.1
GAPDH
1 2 3 4 5 6 7 8 9 10
MCF-10A
d
control
MCT-1
0 3 6 9 12 15 0 3 6 9 12 15 CHX (h)
PTEN
1 0.9 0.7 0.3 0.1 0 0.9 0.8 0.5 0.1 0 0
V5-MCT-1
GAPDH
MDA-MB-231
e
Reminding of PTEN protein (fold)
1.00
0.75
0.50
0.25
0.00
control
MCT-1
t1/2=6.2 h
t1/2=7.6 h
0 5 10 15
Cycloheximide (h)

Figure S2. MCT-1 expression and PTEN deficiency synergistically promote spindle multipolarity and multinuclearity. MCF-10A cells with the indicated expression condition of MCT-1 and PTEN were examined. (**a**) Cells were exposed to nocodazole for 24 h and cultured in the nocodazole-free medium for 1 h. Spindle aster (NuMA staining, red), microtubule structure (α-tubulin staining, green) and nuclear DNA (DAPI, blue) were assessed. (**b**) Cell populations with multiple spindle arrays were quantified. The numbers of cells scored in each cellular context were indicated. (**c**) Cells were exposed to nocodazole for 24 h and released for 1 h. Mitotic regulators p190B, NuMA and p-histone H3 were more expressed in the MCT-1/-PTEN cells than the other cohorts. (**d**) Time-lapse microscopy was conducted. The control/-PTEN cell entered mitosis and divided into two daughter cells. Abortive abscission of the MCT-1/-PTEN cell was observed.

Fig. S2

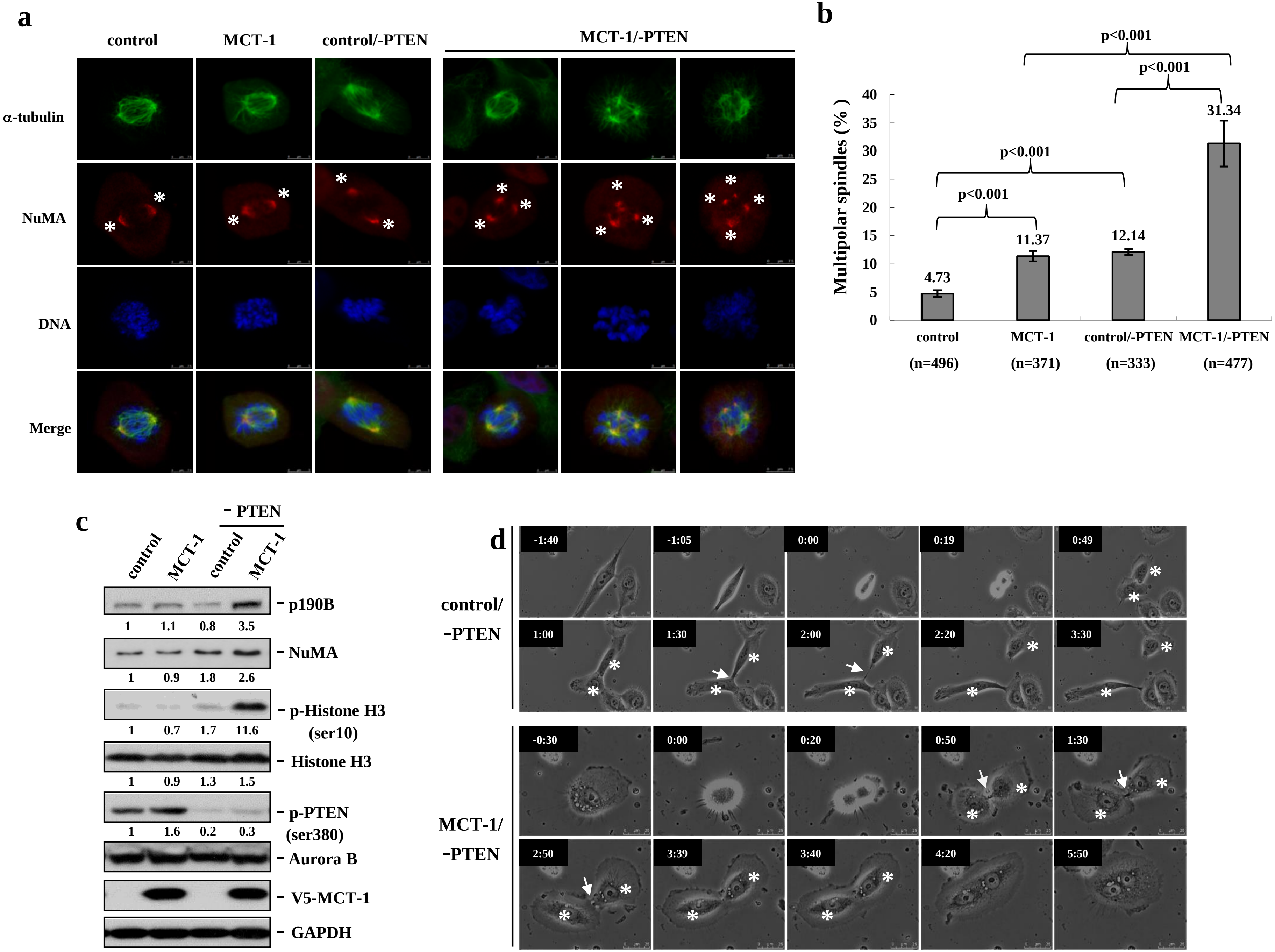

a
control
MCT-1
control/-PTEN
MCT-1/-PTEN
α-tubulin
NuMA
DNA
Merge
b
p<0.001
p<0.001
p<0.001
p<0.001
Multipolar spindles (%)
40
35
30
25
20
15
10
5
0
4.73
11.37
12.14
31.34
control
(n=496)
MCT-1
(n=371)
control/-PTEN
(n=333)
MCT-1/-PTEN
(n=477)
c
- PTEN
control
MCT-1
control
MCT-1
p190B
1 1.1 0.8 3.5
NuMA
1 0.9 1.8 2.6
p-Histone H3
(ser10)
1 0.7 1.7 11.6
Histone H3
1 0.9 1.3 1.5
p-PTEN
(ser380)
1 1.6 0.2 0.3
Aurora B
V5-MCT-1
GAPDH
d
control/
-PTEN
-1:40
-1:05
0:00
0:19
0:49
1:00
1:30
2:00
2:20
3:30
MCT-1/
-PTEN
-0:30
0:00
0:20
0:50
1:30
2:50
3:39
3:40
4:20
5:50

Figure S3. MCT-1 regulates *p190B* gene expression. MDA-MB-468 cells were examined. (**a**) The mRNA levels of *MCT-1*, *p190B* and *p190A* in the vector control and MCT-1-overexpressing cells were analyzed by Q-RT-PCR. Expression levels of *p190B* but not *p190A* was stimulated by ectopic MCT-1 expression. (**b**) The *MCT-1*, *p190B* and *p190A* mRNA levels were analyzed in the cells with (shMCT-1) or without (MOCK) MCT-1 knockdown. Knockdown of MCT-1 suppressed *p190B* gene expression.

Fig. S3

a

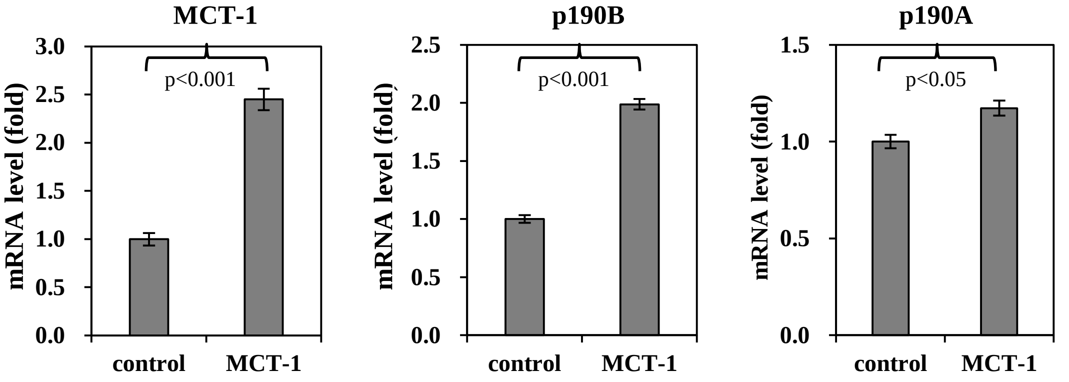
MCT-1
p190B
p190A
3.0
2.5
2.0
1.5
1.0
0.5
0.0
mRNA level (fold)
p<0.001
p<0.05
control
MCT-1

b

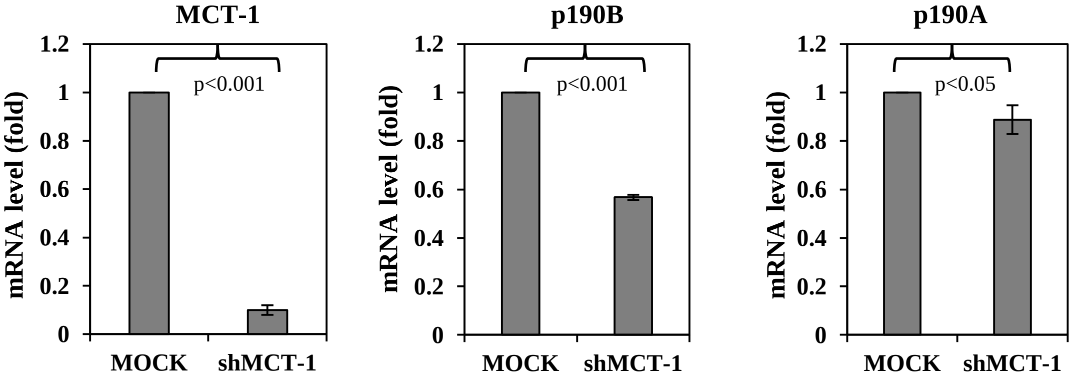
MCT-1
p190B
p190A
1.2
1
0.8
0.6
0.4
0.2
0
mRNA level (fold)
p<0.001
p<0.05
MOCK
shMCT-1

Figure S4. PTEN loss and MCT-1 activation synergistically promotes multinucleation. **(a)** MCF-10A cells with the indicated expression condition of MCT-1 and PTEN were reactivated for 30 min after serum starvation for 24 h. The active Src (tyr416) and p190B (p-tyr) levels were induced and the active RhoA (RhoA-GTP) was suppressed in MCT-1/-PTEN cells. **(b)** Multinuclear frequencies and numbers of cells scored in each group were indicated.

Fig. S4

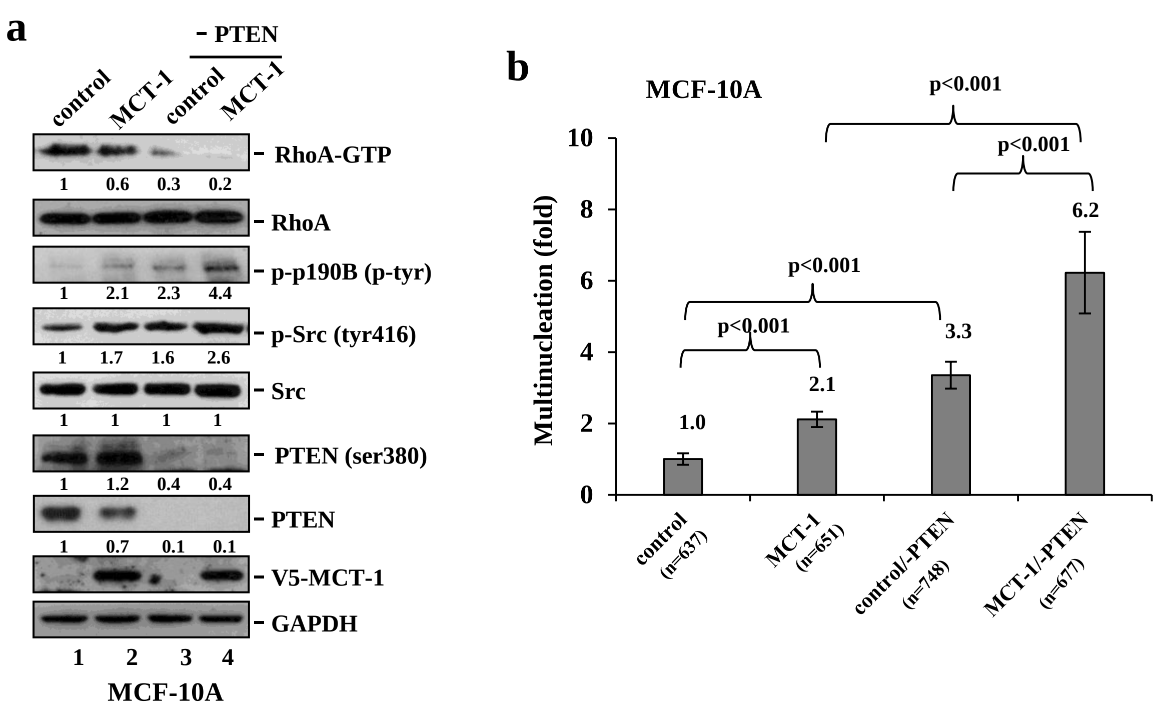
a
- PTEN
control
MCT-1
control
MCT-1
RhoA-GTP
1 0.6 0.3 0.2
RhoA
p-p190B (p-tyr)
1 2.1 2.3 4.4
p-Src (tyr416)
1 1.7 1.6 2.6
Src
1 1 1 1
PTEN (ser380)
1 1.2 0.4 0.4
PTEN
1 0.7 0.1 0.1
V5-MCT-1
GAPDH
1 2 3 4
MCF-10A
b
MCF-10A
Multinucleation (fold)
0
2
4
6
8
10
p<0.001
p<0.001
p<0.001
p<0.001
1.0
2.1
3.3
6.2
control (n=637)
MCT-1 (n=651)
control/-PTEN (n=748)
MCT-1/-PTEN (n=677)

Figure S5. Overexpressing MCT-1 deregulates G2/M mitotic checkpoint. (**a**) After treatment with nocodazole or taxol, cell cycle profiling was examined by flow cytometry. The ectopic MCT-1 cells arrested at the G2/M stage were less than the control cells upon microtubule disruption. **(b)** The G2/G1 ratio upon mitotic arrest was analyzed. Reduced G2/G1 ratio may correspond to the G2/M checkpoint impaired in the ectopic MCT-1 cells.

Fig. S5

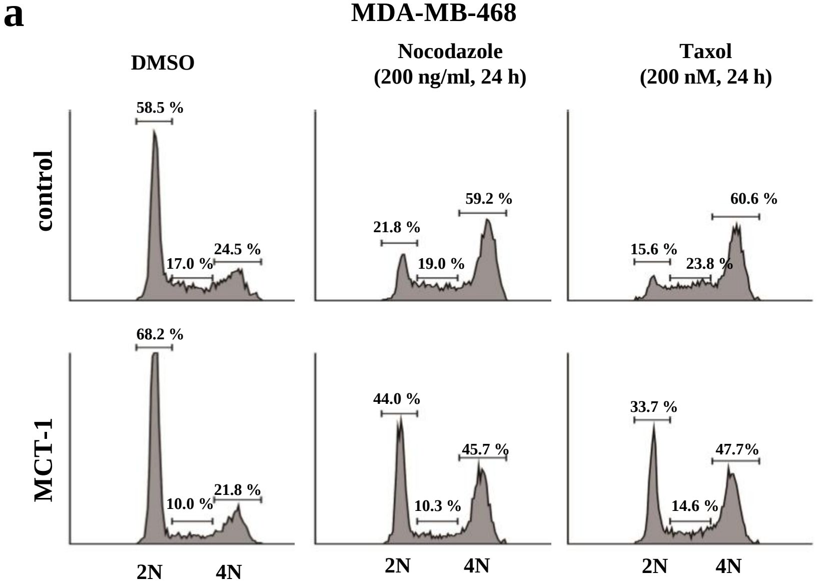

a
MDA-MB-468
DMSO
Nocodazole
(200 ng/ml, 24 h)
Taxol
(200 nM, 24 h)
control
MCT-1
58.5 %
17.0 %
24.5 %
21.8 %
19.0 %
59.2 %
15.6 %
23.8 %
60.6 %
68.2 %
10.0 %
21.8 %
44.0 %
10.3 %
45.7 %
33.7 %
14.6 %
47.7%
2N
4N

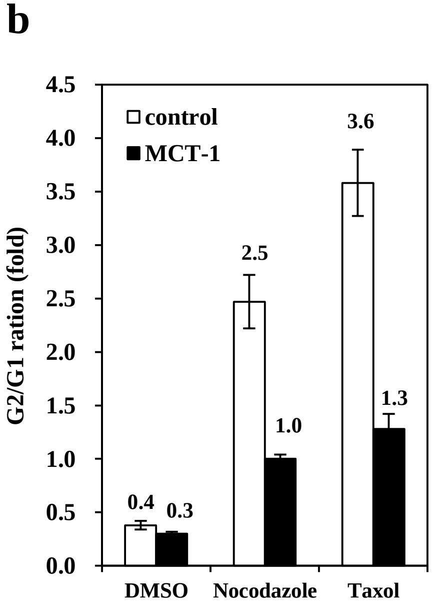

b
control
MCT-1
G2/G1 ration (fold)
4.5
4.0
3.5
3.0
2.5
2.0
1.5
1.0
0.5
0.0
0.4
0.3
2.5
1.0
3.6
1.3
DMSO
Nocodazole
Taxol

Figure S6. Chromosomal abnormalities induced by the overexpression of MCT-1. For the whole genome comparison between the MCT-1 overexpression (test) and control (reference) MCF-10A cells, their genomic DNA samples were individually labeled with Cy3 and Cy5. (a) Chromosome 5p showed a long region with a deletion score of -0.3. (b) Chromosome 7q revealed only a short region with a deletion score of -0.4, followed by a long region with an amplification score of +0.3. (c) Chromosome 18 contained a large area with amplification scores greater than the 0.2 cutoff.

Fig. S6

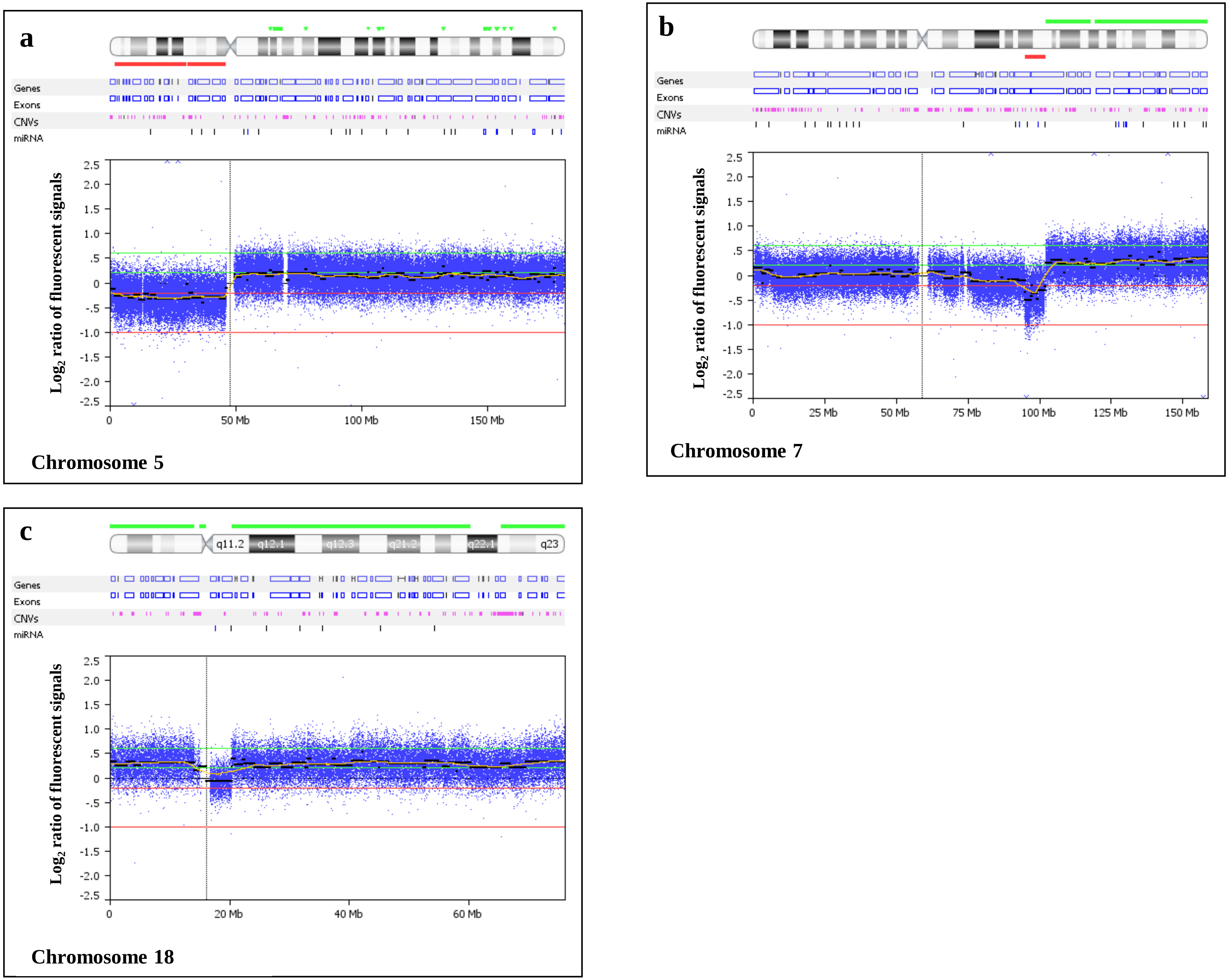
a
Genes
Exons
CNVs
miRNA
Log2 ratio of fluorescent signals
2.5
2.0
1.5
1.0
.5
0
-.5
-1.0
-1.5
-2.0
-2.5
0
50 Mb
100 Mb
150 Mb
Chromosome 5
b
Genes
Exons
CNVs
miRNA
Log2 ratio of fluorescent signals
0
25 Mb
50 Mb
75 Mb
100 Mb
125 Mb
150 Mb
Chromosome 7
c
q11.2
q12.1
q12.3
q21.2
q22.1
q23
Genes
Exons
CNVs
miRNA
Log2 ratio of fluorescent signals
0
20 Mb
40 Mb
60 Mb
Chromosome 18
