# Supplementary material for: MCT-1 expression and PTEN deficiency synergistically promote neoplastic multinucleation through the Src/p190B signaling activation
Source: Oncogene. 2014 May 26;33(43):5109–20. doi: 10.1038/onc.2014.125 (PMC4287651; doi:10.1038/onc.2014.125)
Supplement: Supplementary Materials and Methods [file onc2014125x2.pdf]

Supplementary Materials and Methods

Antibodies

Antibodies (Abs) against PTEN, phospho-PTEN (ser380), histone H3, phospho-histone H3 (ser10), CENP-A, Src, phospho-Src (tyr416), AKT, phospho-AKT (ser473), ERK1/2, phospho-ERK1/2 (thr202/tyr204) and phospho-EGFR (tyr1068) were purchased from Cell Signaling Technology. Abs recognized MCT-1 (2G2) and p190B (EP489Y) were purchased from (GeneTex). Abs against p53 (DO-1) (Santa Cruz Technology), V5 epitope (Invitrogen), GAPDH (Abcam) and phospho-Tyr (4G10) (Millipore) were purchased from different vendors.

Inhibition of protein kinase and proteasome activity

MCF-10A cells were starved for 24 h in DMEM/F12 basal medium (without growth-essential factors and HS) before treatment with 30 μM UO126 (ERK1/2 inhibitor) (Cell Signaling), 50 μM LY294002 (PI3K inhibitor) (Cell Signaling), 100 μM wortmannin (PI3K inhibitor) (Sigma) or 30 μM PP2 (Src inhibitor) (Calbiochem) for 4 h. MCF-10A cells treated with or without each inhibitor were re-activated in regular DMEM/F12 medium for 30 min. MCF-7 cells were starved in serum-free RPMI 1640 medium for 24 h and then incubated with or without 100 μM wortmannin for 4 h followed by activation with 100 nM insulin and 10% FBS for 30 min.

MCF-10A cells were starved for 12 h and incubated with or without MG132 (50 μM) for

another 12 h before serum activation for 30 min. The effects of proteasome inhibition on AKT (ser473), PTEN and p53 were evaluated.

Immunoblot analysis

The active phosphorylation of AKT, PTEN, ERK1/2 and EGFR were studied in the MCF-10A cells activated for 30 min in DMEM/F12 complete medium after starving in DMEM/F12 basal medium for 24 h. The cellular response in late mitotic stage was analyzed by that MCF-10A cells were treated with nocodazole (50 ng/ml) for 18 h, washed with PBS and re-cultured in nocodazole-free medium for 1 h. The phosphorylation of Src, p190B and RhoA were examined when MCF-10A or MDA-MB-468 cells were activated with serum for 30 min after starving for 24 h. The protein samples were analyzed by SDS-PAGE and immunoblotting as previously described (31).

MTT (3-(4, 5-dimethylthiazol-2-yl)-2, 5-diphenyltetrazolium bromide) assay

MCF-10A cells were cultured in a 96-well plate for 24 h, washed twice with PBS and incubated in DMEM/F12 basal medium (without serum and growth factors). Cell growth rate was analyzed using the Cell Proliferation Kit I (Roche) as described previously (31).

Immunofluorescence and fluorescence time-lapse microscopy

The cells were cultured in nocodazole-free media for 30 min after nocodazole treatment for 24 h.

Immunofluorescence study was conducted as described previously (28). The fluorescence images were captured using a Leica TCS NT confocal microscope (Leica) equipped with a 63x objective lens (HCX PLAPO lambda blue 63x NA 1.4 UV) and analyzed by Leica LAS AF software. For time-lapse microscopy, the cells ($1x10^5$) were cultured in a 0.17-mm ibidi μ-dish (ibidi) and incubated with or without Hochest 33342 dye (100 ng/ml) (Invitrogen). The images were recorded at 20- or 30-min intervals for 36 h using a Leica AF6000 LX fluorescence imaging system (Leica) equipped with a 20x objective lens (HCX PLAPO 20x NA 0.7) and analyzed by LASAF software.

Immunoprecipitation assay

Cells were extracted with RIPA buffer (50 mM Tris-HCl, pH 8.0, 150 mM NaCl, 1% NP-40, 0.5% sodium deoxycholate, 0.1% SDS) containing the cocktails of phosphatase inhibitors (Sigma) and protease inhibitors (Sigma). Cell lysates (1 mg) were pre-clarified with 2 μg pre-immune Ab at 4^oC for 1 h and incubated with 50 μl slurry of 50% protein A/G magnetic beads (Millipore) for 30 min. The clarified lysates were reacted with pre-immunized IgG or p190B Ab for 4 h at 4^oC, incubated with 50 μl protein A/G magnetic beads for 4 h at 4^oC and washed by RIPA buffer for 5 times before SDS-PAGE. The immune-reactive proteins were detected by HRP-conjugated EasyBlot anti-mouse or anti-rabbit secondary Ab (GeneTex).

Flow cytometry analysis

MDA-MB-468 cells were treated with 200 ng/ml nocodazole (Sigma) or 200 nM Taxol (Sigma) for 24 h. MCF-10A cells were treated with 50 ng/ml nocodazole for 24 h. Cells were fixed with 70% ethanol for 2 h or stored at -20℃ and stained with 10 μg/ml propidium iodide (PI) (Sigma) in PBS with 0.2 μg/ml DNase-free RNase A (Sigma) at room temperature for 2 h. Cell cycle profiling was analyzed by the BD FACSCalibur flow cytometer (Becton-Dickinson).

RhoA activity assay

RhoA activity was analyzed using the Rho activation assay kit (Millipore). Briefly, MCF-10A and MDA-MB-468 cell lysates were extracted by MLB lysis buffer (10% glycerol, 25 mM HEPES, pH 7.5, 125 mM NaCl, 1% NP-40, 10 mM $MgCl_2$, 1 mM EDTA). The protein extracts (1 mg) were incubated with 20 μl of Rhotekin RBD-GST-conjugated agarose beads for 45 min at 4℃. After washing 3 times with MLB buffer, the beads were dissolved in 2X SDS-PAGE loading buffer, resolved by NuPAGE 4%-12% Bis/Tris gels and detected with RhoA Ab.

Immunofluorescent detection of phosphorylated histone H3

The MDA-MB-468 cells were harvested after 24 h of nocodazole (200 ng/ml) or taxol (200 nM) treatment and fixed in 70% ethanol at -20 ◦C for at least 4 h. After fixation, cells were washed twice with PBS and then washed once with staining buffer (2% FBS in PBS). Cells were stained with 20 μl of Alexa Fluor 647 Rat anti-Histone H3 (ser28) (Becton-Dickinson) in 80 μl of staining

buffer at room temperature for 20 min, washed twice with staining buffer and then incubated with 10 μg/ml propidium iodide (PI) (Sigma) in PBS containing 0.2 μg/ml DNase-free RNaseA (Sigma) at room temperature for 30 min. Cellular fluorescence and cell cycle profiling was measured by using a BD FACSCalibur flow cytometer (Becton-Dickinson).

Cytogenetic study

The MCF-10A cells were mitotic arrested with 0.1 μg/ml colcemid (Calbiochem) for 4 h. Cells were harvested and incubated with pre-warmed 75 mM KCl at 37 ◦C for 40 min. Following hypotonic swelling, fresh fixative reagent (methanol:acetic acid = 3:1) was slowly dropped into cell pellets while gently tapping the tubes. Cell samples were fixed at room temperature for 10 min and re-fixed twice. The fixed samples were dropped onto slides and dry slides at 100◦C for 25 min. The samples were stained with Wright's stain solution (MERCK) for 1 min. The chromosome number were analyzed for at least 100 metaphase cells.

ArrayCGH study and data analysis

DNA samples (test and reference) were obtained from the control and MCT-1-overexpressing MCF-10A cell lines using genomic PUREGENE DNA purification kit (Gentra Systems). All samples were sheared by sonication and labeled with 532 nm-Cy3 and 635 nm-Cy5 dye-modified random primers. Labeled DNA samples of each group were combined and hybridized to a

NimbleGen CGH Human Genome Slide (NimbleGen Systems) using MAUI hybridization System & MAUI Mixer SL Low Tempature hybridization chambers (BioMicro Systems). Afterward, all the array slides were stringently washed, dried, and scanned using a two-color scanning protocol of Axon GenePix Scanner 4000B (Molecular Devices). Data analysis was performed using NimbleScan software V2.2. To identify chromosome segmental aberrations, the resultants were viewed using SignalMap software V1.9 (Roche NimbleGen Inc). Copy number aberrations were verified by using Nexus Copy Number software (BioDiscovery, Inc.).
